# Pathological Implications of Receptor for Advanced Glycation End-Product (*AGER*) Gene Polymorphism

**DOI:** 10.1155/2019/2067353

**Published:** 2019-02-04

**Authors:** Marine Serveaux-Dancer, Matthieu Jabaudon, Isabelle Creveaux, Corinne Belville, Raïko Blondonnet, Christelle Gross, Jean-Michel Constantin, Loïc Blanchon, Vincent Sapin

**Affiliations:** ^1^CHU Clermont-Ferrand, Department of Medical Biochemistry and Molecular Biology, 63000 Clermont-Ferrand, France; ^2^University Clermont Auvergne, CNRS UMR 6293, INSERM U1103, GReD, 63000 Clermont-Ferrand, France; ^3^CHU Clermont-Ferrand, Department of Perioperative Medicine, 63000 Clermont-Ferrand, France

## Abstract

The receptor for advanced glycation end-products (RAGE) is a cell surface transmembrane multiligand receptor, encoded by the *AGER* gene. RAGE presents many transcripts, is expressed mainly in the lung, and involves multiple pathways (such as NF*κ*B, Akt, p38, and MAP kinases) that initiate and perpetuate an unfavorable proinflammatory state. Due to these numerous functional activities, RAGE is implicated in multiple diseases. *AGER* is a highly polymorphic gene, with polymorphisms or SNP (single-nucleotide polymorphism) that could be responsible or co-responsible for disease development. This review was designed to shed light on the pathological implications of *AGER* polymorphisms. Five polymorphisms are described: rs2070600, rs1800624, rs1800625, rs184003, and a 63 bp deletion. The rs2070600 SNP may be associated with the development of human autoimmune disease, diabetes complications, cancer, and lung diseases such as chronic obstructive pulmonary disease and acute respiratory distress syndrome. The rs1800624 SNP involves *AGER* gene regulation and may be related to reduced risk of heart disease, cancer, Crohn's disease, and type 1 diabetes complications. The rs1800625 SNP may be associated with the development of diabetic retinopathy, cancer, and lupus but may be protective against cardiovascular risk. The rs184003 SNP seems related to coronary artery disease, breast cancer, and diabetes. The 63 bp deletion may be associated with reduced survival from heart diseases during diabetic nephropathy. Here, these potential associations between *AGER* polymorphisms and the development of diseases are discussed, as there have been conflicting findings on the pathological impact of *AGER* SNPs in the literature. These contradictory results might be explained by distinct *AGER* SNP frequencies depending on ethnicity.

## 1. *AGER* Overview

### 1.1. RAGE: Structure and Expression

**Figure 1:**
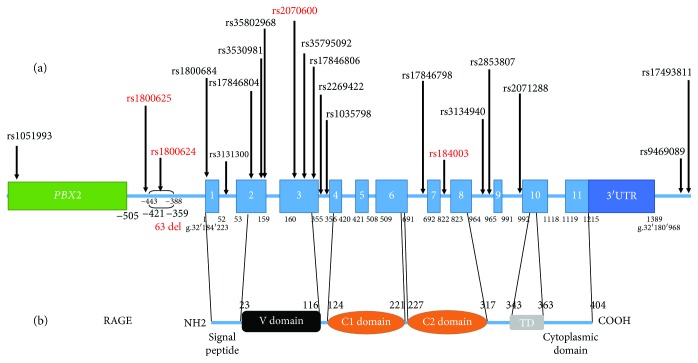
(a) Representation of human predominant RAGE transcript (NM_001136) with principal polymorphisms described for this form to date; polymorphisms in red are the most common ones. (b) Representation of human RAGE protein corresponding to RAGE transcript (NM_001136).

*AGER* (advanced glycation end-product-specific receptor [OMIM_600214]) encodes a cell surface receptor for advanced glycation end-products (RAGE). This gene is located on the short arm of chromosome 6: 6p21.3 [1]. This locus is involved in inflammatory and immune responses and is also the locus of major histocompatibility complex III. The *AGER* gene was identified in 1994 [2]. Its 5′ flanking region from -505 overlaps with the *PBX2* gene [3]. Many alternatively spliced transcript variants encoding different isoforms, as well as non-protein-coding variants, are described for this gene. Ensembl [4] described 15 different transcripts, whereas some papers described up to 19 transcripts [5–7]. The longest isoform (NM_001206929) has 11 exons and encodes a 420-amino acid (AA) protein. The predominant transcript (NM_001136) has 11 exons and encodes a 404 AA protein, 55 kDa [5] (cDNA: 1492 bp, DNA sequence: 4557 bp). The first 22 AA correspond to a signal peptide. Three immunoglobulin-like domains are encoded: a V-type domain (23-116 AA) and two C-type domains (124-221 and 227-317 AA). The protein exhibits only one transmembrane domain (343-363 AA) and has a short highly charged cytosolic tail (364-404 AA) that is critical to intracellular signaling [5, 8, 9] (see Figure 1). The V domain and its closest C domain have a high content of arginine and lysine residues carrying a positive charge; the second C domain has mainly acidic residues and is charged negatively. These three distinct domains explain how RAGE can be a multiligand receptor, with the ability to assemble as a multimer depending on the ligand [10, 11].

Beyond the full-length RAGE (NM_001136), three major isoforms are expressed and represent 90% of detectable transcripts. The N-RAGE (N-truncated RAGE) transcript has an in-frame stop codon in the intronic sequence and starts its initiation at exon 3; therefore, it does not express the V-type immunoglobulin domain. This transcript corresponds to a 303-AA protein (42 kDa), which is transported and localized on the plasma membrane as full-length RAGE. This membrane isoform cannot bind any RAGE ligand, and its biological function is poorly understood to date [5, 12–14]. Two other transcripts are called C-truncated and lack the transmembrane and cytoplasmic domains of the full-length receptor. Another transcript encoding known as sRAGE (soluble RAGE) is formed by proteolytic cleavage of full-length RAGE by a protease, principally ADAM10 (a disintegrin and metalloproteinase domain-containing protein 10), while esRAGE (endogenous secretory RAGE) is encoded through alternative splicing of full-length RAGE and secreted by cells. sRAGE and esRAGE are two soluble C-truncated RAGE proteins which circulate in the blood and other biological fluids. These proteins can bind AGEs (advanced glycation end-products) and other RAGE ligands [14–16]. The C-truncated isoforms are endogenous competitive RAGE inhibitors that do not affect the metabolic pathways and act as decoys [17]. Of note, esRAGE serum levels are two- to fivefold lower than those of sRAGE in healthy subjects and sRAGE (50 kDa) and esRAGE (46 kDa) may not have equivalent biomarker values [14].

RAGE is a member of the immunoglobulin superfamily. It is a multiligand cell surface receptor. RAGE principally binds AGEs (produced through glycation of proteins or lipids after sugar exposure), a polypeptide in-inked with neuronal growth (HMGB1, high-mobility group protein 1 or amphoterin), and members from the S100 family (S100A8, S100A9, S100A11, S100A12, and S100B). RAGE activation after ligand binding increases receptor expression and activation of proinflammatory and procoagulatory pathways, leading, for example, to vascular dysfunction. Several phosphoproteins (NF*κ*B, Akt, p38, and MAP kinases) and adaptors (MyD88, TIRAP, Dock7, and DIAPH-1) are involved in RAGE-associated intracellular pathways [3, 8]. The expression of RAGE isoforms is tissue-specific and varies depending on the ligand, thus explaining the important variability of molecular and cellular answers after RAGE activation. RAGE is highly expressed during embryonic developmental stages, and although it is expressed widely in the whole fetal body (heart, liver, brain, kidney, etc.), RAGE expression culminates in the lung. Under physiological conditions, RAGE is expressed in multiple adult tissues but predominates in the lung, with a weak basal level in general and in cells involved in immune/inflammatory responses in particular. Elevated RAGE levels have been reported in multiple diseases [10, 12, 13, 16–18].

### 1.2. Pathophysiological Implications

RAGE causes an unfavorable proinflammatory state implicated in multiple pathways and diseases: inflammatory diseases, rheumatic or autoimmune diseases, infectious diseases, diabetes, metabolic syndrome and its complications, obesity, insulin resistance, hypertension, atherosclerosis, neurological diseases such as Alzheimer's disease, cardiovascular diseases, pulmonary diseases such as chronic obstructive pulmonary disease (COPD), and cancer. Genetic variations of RAGE should be considered as responsible or co-responsible for the development of diseases [3, 17, 19–22]. In addition, combining RAGE-circulating protein levels and *AGER* polymorphisms may be a useful clinical tool for risk prediction of vascular diseases [18]. Indeed, sRAGE could be a valuable biomarker in many pathological states. It increases in patients with decreased renal function, but decreases in diabetic complications and coronary artery disease, and elevated levels of sRAGE are often associated with end-stage disease [14]. Recently, sRAGE was demonstrated as a new biomarker for lung cancer and RAGE could be a potential therapeutic target in Alzheimer's disease [23, 24]. The genetic background of RAGE demonstrated that some gene polymorphisms are implicated in various pathological states, for example, diabetes complications, amplification of the inflammatory response, non-small cell lung cancer, gastric cancer, or breast cancer [25]. In this review, we focused on principal *AGER* polymorphisms reported in the literature, in particular on the five main *AGER* polymorphisms described (rs2070600, rs1800624, rs1800625, rs184003, and a 63 bp deletion). Some *AGER* polymorphisms correlate with circulating levels of RAGE isoforms [13, 22, 26]. In this review, we chose to focus on general frequency in the CEU population, who are Utah residents with Northern and Western European ancestry, and in the EAS population, who are East Asians (http://phase3browser.1000genomes.org/), because frequency diversities exist in the literature, depending on the ethnicity and the study population [27]. All polymorphisms described in this work and their potential pathological implications are reported in Table 1.

## 2. rs2070600 NM_001136.4:c.244G>A p.Gly82Ser Polymorphism

This SNP (single-nucleotide polymorphism), often referred to as Gly82Ser or G82S, is the most described SNP within the *AGER* gene. It is reported in the Human Gene Mutation Database (HGMD) (http://www.hgmd.cf.ac.uk/ac/index.php) as being associated with microangiopathy in type 2 diabetes [28]. In the general population, the G allele frequency is 92.8% and the A allele frequency is 7.2%. In the CEU population, the G and A allele frequencies are 92.4% and 7.6%, respectively; in the EAS population, they are 78.1% and 21.9%, respectively (1000 Genome data). The rs2070600 SNP or Gly82Ser polymorphism is located in exon 3 of the *AGER* gene. The rs2070600 SNP probably emerged after migrations from Africa, reaching the highest frequencies in Asia [29]. It is a missense variation inducing the substitution of glycine by serine at codon 82 within the RAGE protein. It involves the formation of an *AluI* restriction site that has facilitated its exploration [30]. Exon 3 is a putative site of ligand binding. The rs2070600 SNP is located in the ligand-binding V domain of *AGER*, suggesting a possible influence of this variant on *AGER* function [31]. To be more specific, rs2070600 is located in the second *N*-glycosylation motif and promotes glycosylation of RAGE, which modifies the RAGE ligand-binding structure and induces an increase in RAGE affinity for ligand AGEs [25, 32]. Functional studies of rs2070600 have found that it affects the structure of the receptor protein, influencing its cleavage by some proteases. The rs2070600 SNP has been reported to decrease proteolysis of RAGE [33]. The rs2070600 SNP is associated with changes in blood AGEs and sRAGE levels. The 82Gly/Gly genotype was reported as strongly associated with higher plasma levels of sRAGE than 82Gly/Ser or 82Ser/Ser genotypes. Heterozygote carriers of rs2070600 had lower sRAGE levels than wild-type carriers. The homozygote carriers of rs2070600 are rare and exhibit lower sRAGE levels as well. These results were observed in different studies, in particular in those of German and Korean cohorts. This association may be explained by a decrease in RAGE proteolysis [33–35]. In addition, healthy homozygote carriers of rs2070600 showed higher AGE serum levels, insulin resistance, plasma TNF-alpha, serum CRP, and 8-epi-prostaglandin F (2alpha) blood concentrations than did heterozygote carriers or wild-type carriers [35]. Homozygotes for the minor allele had higher risk factors for cardiovascular disease, such as low sRAGE levels, inflammation, oxidative stress, and insulin resistance, compared with those bearing at least one G allele. The rs2070600 SNP enhances the stimulation of RAGE by its ligands and induces a proinflammatory signal that stimulates mechanisms underlying inflammatory diseases [33, 35, 36].

Many studies have focused on the implications of rs2070600 in different diseases such as inflammatory diseases, cancer, coronary artery disease, lung diseases, or myocardial infarction, with some conflicting results. The rs2070600 SNP could be relevant in human autoimmune diseases. rs2070600 has been shown to be more frequent in rheumatoid arthritis patients [37]. A decrease in circulating sRAGE has also been shown in children during active autoimmune diseases, such as Kawasaki disease or systemic onset juvenile idiopathic arthritis [38]. The rs2070600 SNP was assessed in diabetic patients with controversial results. An Indian study on type 2 diabetes has shown significant associations between rs2070600 and diabetic retinopathy [39]. Significant associations between rs2070600 with diabetic retinopathy were also reported in Asian Indians and Asian Chinese with type 2 diabetes [40]. A study in Ashkenazi or Sephardic Jewish patients with type 1 or type 2 diabetes established that rs2070600 was associated with the risk of developing diabetic nephropathy [41]. However, this association also seems true in an Asian population. [42]. Another meta-analysis has highlighted a significant association of rs2070600 with the risk of diabetic nephropathy development [43]. The rs2070600 SNP was associated with skin manifestations of microangiopathy and with psoriasis vulgaris in Czech type 2 diabetic patients, compared to the control. The development of dermatoses in subjects with a predisposition to glucose intolerance could be influenced by rs2070600 [28]. An analysis of patients with type 1 diabetes from the Finn Diane cohort has found that rs2070600 could predict an increased risk of type 1 diabetes, with an association with decreased circulating sRAGE levels [38]. Another study showed the same result, although carriers with type 1 diabetes were younger at the time they were diagnosed [22]. These studies have reported that *AGER* polymorphisms were associated with diabetes complications. However, other studies did not find any association with diabetes [44]. A study in a Boston cohort did not find any association between type 2 diabetes, insulin resistance, and *AGER* polymorphisms including rs2070600, even with haplotype analysis [45]. In a Brazilian sample, rs2070600 was not associated with either type 1 or type 2 diabetes and there was a very low frequency of rs2070600 in an African Brazilian population [46]. A meta-analysis did not highlight any significant association between this *AGER* polymorphism and type 2 diabetes, diabetic retinopathy, or diabetic nephropathy risks [47]. A genome-wide association (GWA) study of Asian type 2 diabetic patients revealed an association of *AGER* variants and plasma sRAGE levels, which themselves were associated with diabetic kidney disease. But they did not find any causal link between renal function and rs2070600, even if rs2070600 was associated with sRAGE levels [48]. The lack of a significant association between rs2070600 and diabetic retinopathy has been reported in both Malaysian and Japanese populations [40]. A meta-analysis did not show any significant association between rs2070600 and increased risk of diabetes [49]. The ethnicity might influence the association between rs2070600 and diabetic diseases and its complications, explaining these previous contradictory results.

The association of SNP rs2070600 with an increased risk of cancer development has been evaluated. Some studies have confirmed such an association, such as a study on gastric cancer in a Chinese population, another on epithelial ovarian cancer also in a Chinese population, and another on non-small cell lung cancer in which rs2070600 was associated with a decreased response to chemotherapy and worse prognosis in non-small cell lung cancer [32]. A meta-analysis has shown that rs2070600 frequency was positively related with risk of lung cancer but not with breast cancer [50]. Two meta-analyses confirmed that rs2070600 was significantly associated with cancer risk, especially lung cancer, and with variations of sRAGE levels [51, 52]. Another meta-analysis has revealed that rs2070600 was significantly associated with increased risk of cancer [53], albeit a Czech study established that rs2070600 was associated with lower sRAGE levels. RAGE was implicated in the pathogenesis of pancreatic cancer and its metastatic process, revealed by an *in vitro* experiment. In addition, sRAGE was associated with autoimmune pancreatitis, chronic pancreatitis, pancreatic cancer, and intraductal papillary mucinous cancer of the pancreas. But they did not find any significant differences in allelic and genotype rs2070600 frequencies among patients with pancreatic cancer, diabetes mellitus, and healthy controls, even if rs2070600 was associated with sRAGE levels [25]. Furthermore, a Czech study did not find any association between the presence of rs2070600 and clear cell renal cancer. But RAGE is one of the key factors accelerating tumor progression and metastasis in various types of cancers, including clear cell renal cancer [32].

RAGE is abundantly expressed in the lung. To characterize RAGE expression in the lung, some authors assessed fetal lung samples and found that the mRNA of *AGER* increased with increasing gestational age during human lung development [54]. *RAGE* expression is most abundant in the lung, and its expression in respiratory epithelial cells varies during lung morphogenesis. RAGE has been shown to play a critical role in this process to obtain a mature, functional lung [55]. RAGE might have physiological roles in the lung, and polymorphisms in *AGER* can be associated with lung diseases [56, 57]. A study reported that SNP in *AGER* was associated with pulmonary function in a Chinese Han population [58]. Many GWA studies had associated this locus with lung function and with COPD susceptibility [54, 59]. The rs2070600 SNP was mostly studied. In several studies, a strong association with forced expiratory volume in one second (FEV_1_) and with FEV1/forced vital capacity (FVC) was found. In addition, this association was confirmed in a large GWA study in individuals with European ancestry. A reduction of FEV_1_/FVC is a characteristic of obstructive lung diseases such as asthma. However, rs2070600 did not show any association with adult asthma in another study [60]. sRAGE was positively correlated with FEV_1_ in patients with COPD, a condition that was associated with lower sRAGE levels. The rs2070600 SNP correlated with reduced FEV_1_. The FVC was higher, and the FEV_1_/FVC ratio was lower in children with rs2070600 than in those without rs2070600. In smokers, rs2070600 was associated with lower FEV_1_ and FEV_1_/FVC and lower serum sRAGE levels [54, 55, 57, 61–64]. In these studies, rs2070600 was associated with COPD susceptibility but not with lung cancer [61, 65]. Indeed, rs2070600 was found more frequently in COPD patients than in healthy controls and homozygous carriers developed COPD more frequently. rs2070600 was associated with severe COPD in a study of Caucasian smokers from Poland [66]. Moreover, rs2070600 was also associated with COPD in a Chinese population. The rs2070600 SNP associated with COPD-related pulmonary function might involve an exacerbated inflammatory response in the lung [58]. The rs2070600 SNP was also associated with circulating sRAGE levels. Lower circulating sRAGE levels were associated with emphysema and COPD severity [67]. In a study, rs2070600 was also significantly associated with the amount of emphysema among European Americans and African Americans [62]. However, a study did not find any significant association between rs2070600 and COPD but showed an association between rs2070600 and FEV_1_/FVC [63]_._ In healthy adult mice and humans, *AGER* was highly expressed in the lung and its absence may be implicated in the pathogenesis of idiopathic pulmonary fibrosis. RAGE signaling is involved in host defense, inflammation, and tissue remodeling, which could explain the association of RAGE with an accelerated decline in pulmonary function with age [64]. The rs2070600 SNP was not associated with increased risk of idiopathic pulmonary fibrosis development. However, sRAGE levels were lower among adults with idiopathic pulmonary fibrosis than in control subjects. Moreover, rs2070600 was associated with lower sRAGE levels among adults with idiopathic pulmonary fibrosis, and lower plasma sRAGE levels might be a biological measure of disease severity in this setting [57, 68]. Another study found that the frequency of rs2070600 was significantly higher in patients with idiopathic pulmonary fibrosis. In addition, rs2070600 and a diagnosis of idiopathic pulmonary fibrosis were independently associated with reduced serum levels of sRAGE during acute exacerbations of idiopathic pulmonary fibrosis and were independent predictors of 5-year survival in these patients. In this context, sRAGE could have a prognostic value. A study in a high-risk population of patients admitted to intensive care units reported that rs2070600 was associated with increased risk of developing acute respiratory distress syndrome (ARDS) and with higher plasma sRAGE levels. Plasma sRAGE levels and rs2070600 could therefore be predictive of ARDS development [56, 59, 69].

Only a few studies have explored the links between rs2070600 and cardiovascular diseases. A meta-analysis reported that rs2070600 was not associated with cardiovascular diseases [26]. Two other meta-analyses did not highlight any association between rs2070600 and coronary heart diseases [70, 71]. In the Atherosclerosis Risk in Communities study, rs2070600 was not significantly associated with death, coronary heart disease, diabetes, heart failure, or chronic kidney disease even if rs2070600 was associated with an approximate 50% reduction in sRAGE levels [72]. Moreover, a meta-analysis assessed the relationship between the risk of cardiovascular disease, various RAGE isoforms, and rs2070600; sRAGE levels were nonsignificantly lower in patients with coronary artery disease than in controls but were lower in patients with coronary artery disease who had Caucasian ancestry. Circulating esRAGE levels were remarkably lower in coronary artery disease patients, as well as in subgroups with or without diabetes mellitus and without renal disease. Circulating esRAGE might therefore be considered as a powerful negative predictor for the development of coronary artery disease. The rs2070600 SNP may contribute to coronary artery disease development in patients with diabetes mellitus or renal disease, but this contribution is probably dependent on the ethnicity. The risk of cardiovascular disease was associated with rs2070600 in Eastern Asians but not in Caucasians. It has therefore been hypothesized that diabetes mellitus and/or renal disease might favor the occurrence of coronary artery disease through the inheritance of genetic defects leading to the transcriptional activation of *AGER* [27]. For example, a meta-analysis has suggested an association between rs2070600 and the risk of coronary artery disease and ischemic stroke only in the Chinese population [73].

The rs2070600 SNP was studied for a potential correlation with primary open-angle glaucoma because of its potential role in oxidative stress, but no significant association has been reported [74]. In a Chinese cohort, rs2070600 was associated with Alzheimer's disease and with its early onset. Moreover, plasma sRAGE levels were lower in Alzheimer's disease patients than in controls. The rs2070600 SNP was associated with lower sRAGE levels and with faster cognitive deterioration. These findings suggest that rs2070600 is probably a risk factor for the early onset of Alzheimer's disease [75]. Another analysis from a Chinese cohort showed a higher prevalence of rs2070600 in early-onset Alzheimer's disease patients [76, 77]. Moreover, this result was also found in another study in which rs2070600 was associated with increased risk of Alzheimer's disease [78]. Finally, in a cohort of schizophrenic patients, rs2070600 was associated with decreased esRAGE levels. Patients with schizophrenia have lower esRAGE and sRAGE levels than those without schizophrenia, but no association was found between rs2070600 and schizophrenia itself [79]. However, in another study, the presence of rs2070600 was associated with higher psychoticism factors. In addition, rs2070600 was associated with an increased risk of schizophrenia and was possibly associated with earlier onset. It has therefore been hypothesized that rs2070600 increased RAGE signaling, leading to increased secretion of inflammatory mediators, which in turn might influence mental brain functions through developmental processes and/or neurotoxicity [33].

## 3. rs1800624 NM_001136.4:c.-388T>A Polymorphism

This SNP is among the five most described *AGER* SNPs and is more often referred to as -374T>A in the literature. It is reported in the HGMD (http://www.hgmd.cf.ac.uk/ac/index.php) as being associated with reduced risk of heart disease. The T allele frequency in the general population is 85% and 15% for allele A. The T allele frequency in the CEU population is 80.8% versus 19.2% for allele A. The T and A allele frequencies in the EAS population are 86.3% and 13.7%, respectively (1000 Genome data). The rs1800624 SNP (or -374T/A polymorphism) is located in the promoter region of the *AGER* gene. It is hypothesized that this SNP has an old origin in Africa [29, 80, 81]. The rs1800624 SNP increases the transcriptional activity of *AGER in vitro* by influencing the binding affinity of the transcription factor site [32, 37, 82]. This suggests that rs1800264 is involved in *AGER* gene regulation and influences the pathogenesis of inflammatory diseases or diabetes-related vascular complications [83, 84]. The rs1800624 SNP is associated with a threefold increase in *AGER* expression *in vitro*, including sRAGE expression. sRAGE serves as an endogenous antagonist by neutralizing proinflammatory ligands that are in turn unable to activate inflammatory pathways [85]. Absolute or strong linkage disequilibrium, depending on the studies, was found between promoter polymorphisms: rs1800625 (−429T>C), rs1800624 (−374T>A), and a 63 bp deletion [29]. Only a few haplotype analyses are reported in the literature, with many studies focusing on the pathological role of rs1800624 in diseases such as Crohn's disease, cancer, coronary artery disease, and myocardial infarction, but with some conflicting results [79].

The rs1800624 SNP was described in different types of cancer. The rs1800624 SNP frequency was positively related to an increased risk of breast and lung cancer development in a meta-analysis [50], and another study has reported that rs1800624 was increased in non-small cell lung cancer in particular [29]. The homozygous carrier of the major allele has a significantly reduced risk for non-small cell lung cancer, a finding probably due to the downregulation of RAGE, which is considered as a critical step in the formation of lung tumors [32]. However, rs1800624 was not associated with increased risk of pancreatic cancer development [86]. Many studies have focused on haplotype analyses, considering four main polymorphisms (rs1800625, rs1800624, rs2070600, and rs184003); in a study evaluating the impact of the major allele of rs1800624, the haplotype was significantly associated with increased risk of breast cancer, whereas a haplotype containing the minor allele was less frequent in patients with breast cancer than in controls [87]. Another study did not find any significant association between the presence of rs1800624 and breast cancer [88]; a meta-analysis found no significant association between rs1800624 and increased risk of cancer but instead reported decreased susceptibility to breast cancer along with increased susceptibility to lung cancer [52]. In contrast, a meta-analysis revealed that rs1800624 was significantly associated with reduced risk of cancer [53].

The rs1800624 SNP has often been studied in cohorts of diabetic patients, with a particular focus on their risk of developing cardiovascular diseases. An analysis of patients with type 1 diabetes from the Finn Diane cohort reported that rs1800624 was more often found in type 1 diabetic patients than in controls [38]. A study also suggested a possible association between the presence of rs1800624 and corrected insulin levels 30 minutes after an oral glucose load [45], while another study found an association between the homozygous AA genotype and lower albumin excretion in patients with type 1 diabetes and poor metabolic control. In type 1 diabetic patients, rs1800264 was more frequent in patients with diabetic nephropathy and diabetic retinopathy than without. However, the TT genotype was more frequent in diabetic nephropathy in type 2 diabetic patients than without [89]. The rs1800264 SNP was explored in Malaysian type 2 diabetic patients with or without retinopathy; the SNP frequency in these subgroups did not differ significantly from controls. Two studies in Caucasian populations found that rs1800264 was associated with increased incidence of proliferative retinopathy; however, studies in an Asian Chinese population found contradictory results [84]. In healthy infants, the minor allele was associated with higher plasma glucose and insulin resistance [90]. The A allele was more frequent in type 1 diabetic patients with diabetic nephropathy than without and associated with increased risk of sight-threatening retinopathy and decreased risk of macrovascular complications in type 2 diabetic patients, but decreased risk of macrovascular complications in type 1 diabetic patients [90]. A meta-analysis found no association between rs1800624 and diabetic nephropathy [91] or an increased risk of developing diabetes [49].

Indeed, two other meta-analyses suggest that rs1800624 might be a protective factor for macrovascular complications in type 2 diabetic patients in general [32] and in Caucasian type 2 diabetic patients in particular [92]. The rs1800624 SNP was associated with lower severity of coronary artery disease in type 2 diabetes [29] and with a decreased risk of ischemic heart disease in African Brazilian type 2 diabetic patients [93]. The rs1800264 SNP was also associated with decreased risk of cardiovascular diseases in the general population and in diabetic patients [89]. In two Dutch studies including individuals with normal glucose metabolism, the −374A allele was protective against higher blood pressure and arterial stiffness, whereas in individuals with impaired glucose metabolism or type 2 diabetes, the relationship was inverse [94, 95]. A study also found that among nondiabetic Italians, homozygous carriers of the minor allele had decreased risk of coronary artery disease [96], thus supporting growing evidence that the −374A allele could have a protective effect against cardiovascular diseases [93, 97]. The rs1800264 SNP conferred protection against the development of chronic heart diseases, ischemic heart disease, atherosclerosis, and the severity of cardiovascular diseases in diabetic patients [98, 99]. But a meta-analysis had demonstrated that rs1800624 was associated with coronary artery disease especially among Caucasian type 2 diabetic patients [71]. A protective effect of rs1800624 against diabetic retinopathy in type 2 diabetic patients was identified in a meta-analysis [42]. On the other hand, a study on Caucasian Brazilians with type 2 diabetes did not find any association between the presence of the A allele and diabetic retinopathy, diabetic nephropathy, or ischemic heart disease [93], while other reports did not find any association between rs1800264 and cardiovascular diseases or diabetes [85]. No association was found between rs1800264 and the presence of heart failure in unselected patients and between rs1800264 and higher mortality in those with heart failure [94]. The presence of the -374A allele was also associated with increased risk of multivessel disease. However, rs1800264 SNP was not associated with coronary artery disease severity in nondiabetic Han Chinese [82]. In a Caucasian and African Brazilian cohort with left ventricular systolic dysfunction, rs1800264 was not associated with the susceptibility to and prognosis of heart failure, compared to controls [94]. In addition, two meta-analyses found no association between rs1800624 and coronary heart disease [26, 70]. In contrast, only one study reported rs1800264 as a risk factor for coronary artery disease in Turkish patients with diabetes [100].

As RAGE is involved in the inflammatory pathway, rs1800264 has been extensively studied in patients with inflammatory diseases. After kidney transplantation, no difference was observed between patients with rs1800264 and those without the SNP in terms of histological findings, chronic allograft nephropathy, or subclinical rejection [89]. The rs1800264 SNP was similarly distributed between patients with Kawasaki disease and healthy controls [83]. The rs1800624 SNP was associated with decreased risk of Crohn's disease in a Chinese [101] and German population [102], but no association of rs1800264 with Crohn's disease was observed in an American cohort [102]. In patients with major trauma, rs1800264 was not associated with a higher risk of sepsis and multiple-organ dysfunction development [103]. Invasive aspergillosis is a major threat to the successful outcome of hematopoietic stem cell transplantation. The rs1800264 SNP was associated with increased susceptibility to invasive aspergillosis when presented in either hematopoietic stem cell graft recipients or donors [104]. Finally, rs1800624 was less prevalent in patients with systemic lupus erythematosus and lupus nephritis than in controls. In addition, the major allele of rs1800624 was associated with disease severity and response to treatment in patients with lupus nephritis [105].

## 4. rs1800625 NM_001136.4 : c.-443T>C Polymorphism

This SNP is among the five most described *AGER* SNPs and is more often referred to as -429T>C in the literature. It is reported in the HGMD (http://www.hgmd.cf.ac.uk/ac/index.php) as being associated with diabetic retinopathy. The T allele frequency in the general population is 86.3% and is 13.7% for allele C. The T allele frequency in the CEU population is 81.8% versus 18.2% for allele C. The T and C allele frequencies in the EAS population are 90.4% and 9.6%, respectively (1000 Genome data). The rs1800625 SNP or -429T/C polymorphism is located upstream to rs1800624 in the *AGER* gene promoter. It is hypothesized that this SNP has an ancient origin in Africa and was subsequently distributed among populations by migration [29, 32]. The rs1800625 SNP was found to increase the transcription of *AGER in vitro.* It involves an increase in RAGE expression, which might influence the pathogenesis of inflammatory diseases [14, 32, 83, 106, 107]. The level of increased expression of RAGE is around twofold *in vitro* [85]. There is a higher expression of sRAGE and esRAGE in the presence of rs1800625 [18]. Also, rs1800625 is associated with increased AGEs [36]. However, in contrast to rs1800624, no clear difference was observed in transcription factor binding in the presence or absence of rs1800625 [108]. Absolute or strong linkage disequilibrium (depending on the studies) was found between the promoter polymorphisms rs1800625 (−429T>C), rs1800624 (−374T>A), and a 63 bp deletion, as described by the literature with haplotype analysis [29]. Many studies on different pathologies (inflammatory disease, cancer, coronary artery disease, lung disease, and myocardial infarction) focused on rs1800625, but sometimes with conflicting results.

The rs1800625 SNP was studied in different types of cancer. RAGE is implicated in the pathogenesis of pancreatic cancer and its metastasizing. It is expressed concordant to the metastatic ability of human pancreatic cancer cells as demonstrated in an *in vitro* experiment, but rs1800625 was not involved in the pathogenesis [25]. However, in a study, rs1800625 was positively correlated with the susceptibility to lung cancer, but not with breast cancer [50]. In a haplotype analysis including rs1800625, rs1800624, rs2070600, and rs184003, it played a role in development of breast cancer [87]. A study on renal cancer had shown that high aggressiveness of the tumor in clear cell renal cancer was associated with the presence of the C allele [32]. Two meta-analyses did not show a significant association between rs1800625 and cancer risk [52, 53].

No significant difference in the prevalence of rs1800625 was found between patients with Kawasaki disease and healthy controls [83]. rs1800625 was not associated with Crohn's disease in Chinese [101] or in German and American patients, compared to controls [102]. After kidney transplantation, no difference was found in the presence of rs1800265 between patients with or without normal histological findings, chronic allograft nephropathy, and subclinical rejection [89]. However, rs1800265 was significantly associated with sepsis morbidity and development of multiple-organ dysfunction in Chinese Han patients with major trauma, a population in which it could be used as a risk estimate [103]. The rs1800625 SNP was significantly more prevalent in systemic lupus erythematosus and lupus nephritis patients than in controls. In addition, rs1800625 was associated with disease severity and response to treatment in lupus nephritis patients [105]. However, the rs1800625 SNP was not associated with schizophrenia [79].

There were numerous studies of SNP rs1800625 in diabetic patients, with conflicting results. The rs1800625 SNP was significantly increased in type 2 diabetes subjects with retinopathy, compared to those without [32, 108]. A haplotype analysis including rs1800625 and rs3134940 (2184A/G) revealed that the haplotype increased (+65%) the risk of developing diabetic nephropathy and was associated with its early onset [109], findings which were confirmed by another study [110]. The rs1800625 SNP was associated with chronic renal failure in an Asian Indian cohort with type 2 diabetes [98]. The rs1800625 SNP was also associated with the development of insulin resistance [17, 107], and in a Brazilian population there was a twofold increase in the incidence of rs1800625 when subjects were diabetic [111]. An analysis in infants with type 1 diabetes from the Finn Diane cohort also reported that rs1800625 was more prevalent in type 1 diabetic patients than in controls [38]. In diabetic subjects, rs1800625 was associated with higher HbA1c levels, with an increased incidence of type 1 diabetes, and rs1800625 was linked to insulin resistance in healthy subjects [18]. Another study has established that a higher maximal hemoglobin A1c concentration was associated with rs1800625 carriers among patients with diabetes [112]. A meta-analysis found an association of rs1800625 with the development of diabetic nephropathy in patients with type 2 diabetes [91]. However, some studies described controversial results. For example, in a cohort of patients with gestational diabetes, the rs1800625 SNP was not associated with the phenotype [85]. In Brazilians with type 2 diabetes, rs1800625 was not associated with diabetic retinopathy, diabetic nephropathy, or ischemic heart disease [93]. In another study in type 2 diabetic patients, rs1800625 was again not associated with the phenotype or with insulin resistance, even in haplotype analysis [45]. A meta-analysis also supports the lack of association between rs1800625 and increased risk of diabetic retinopathy [113]. A study in a Malaysian population reported that rs1800625 frequency was not different among patients with or without diabetes and diabetic retinopathy [84]. No significant association between this RAGE polymorphism and increased risks of type 2 diabetes, diabetic retinopathy, or diabetic nephropathy development was found in different meta-analyses [42, 43, 47, 49].

The association of rs1800625 with cardiovascular risk has been evaluated, with again controversial results. In a meta-analysis, rs1800625 was associated with higher levels of circulating forms of RAGE (sRAGE and esRAGE) and an increased risk of developing coronary artery disease, especially in diabetic patients. Circulating esRAGE levels were remarkably lower in coronary artery disease patients, in whom it could be used as a negative predictor for the development of coronary artery disease. The authors had hypothesized that diabetes and/or renal disease might precipitate the occurrence of coronary artery disease via the inheritance of genetic defects leading to the transcriptional activation of *AGER*, thus being correlated with patient ethnicity [27]. Another study in individuals who developed atherothrombotic events (incident myocardial infarction or ischemic stroke) reported that rs1800625 was associated with reduced risk of incident myocardial infarction and could have a protective role in atherothrombosis [114]. A haplotype analysis including rs2070600, rs1800624, and rs1800625 found the same result, but even in the presence of the T allele, there was an association of the haplotype with reduced risk of ischemic stroke [18]. A meta-analysis had reported that rs1800625 was associated with coronary artery disease in patients with diabetes mellitus or renal disease [27]. A Chinese study had noted that rs1800625 was associated with a risk of developing coronary artery disease [115]. On the other hand, within different meta-analyses, rs1800625 has been reported to have no association with coronary artery disease [26, 70, 71, 94]. Even so, rs1800625 was not associated with blood pressure and had no effect on the susceptibility to hypertension [116]. The rs1800625 SNP was studied in an ischemic stroke cohort, and it was not associated with any risk, even with small-vessel disease [117].

A study had shown an association between lung disease severity and the presence of rs1800625 in European patients. The rs1800625 SNP increases RAGE expression, which in turn increases lung inflammation. Therefore, RAGE could be an interesting biomarker of lung disease severity [106].

## 5. A 63 bp Deletion NM_001136.4:c.-421_-359del Polymorphism

This SNP is one of the five most described SNPs of the *AGER* gene. This rare variant is also known in the literature as a 63 bp deletion that corresponds to a -421 to -359 deletion. It is reported in the HGMD (http://www.hgmd.cf.ac.uk/ac/index.php) as being associated with reduced survival from heart disease in patients with diabetic nephropathy. This variant is located in the *AGER* gene promoter; it overlaps with rs1800624 and is downstream from rs1800625. It is known to increase the transcriptional activity of *AGER* [37]. A study has explored the impact of this variant on the risk of cardiovascular disease in Chinese subjects with type 2 diabetic nephropathy in which the 63 bp deletion was associated with decreased cardiovascular event-free survival [118]. The 63 bp deletion seems in linkage disequilibrium with rs1800624 and rs1800625 [29]. A report on heart failure patients of different origins has shown that the 63 bp deletion was less frequent in African Brazilian patients than in controls, without any effect on survival when present [94]. Furthermore, this 63 bp deletion was not associated with breast cancer in a study cohort from Iran [88].

## 6. rs184003 NM_001136.4:c.822+49G>T Polymorphism

This SNP is more commonly known as 1704G/T. The G allele frequency in the general population is 84.4% (15.6% for allele T). The G allele frequency in the CEU population is 94.9% versus 5.1% for allele T. The G and T allele frequencies in the EAS population are 85.4% and 14.6%, respectively (1000 Genome data).

In a study in which rs2071288 and rs17846798 were also evaluated, rs184003 was explored in a specific haplotype; the three SNPs were in perfect linkage disequilibrium, and the haplotype was associated with a decrease in esRAGE levels in subjects with schizophrenia [79]. In another study, rs184003 was not associated with blood pressure and did not influence the susceptibility to hypertension [116]. The rs184003 SNP was associated with a higher risk of developing coronary artery disease in a Chinese study [115]. However, rs184003 was associated with increased risk of breast cancer development in haplotype analysis including rs1800625, rs1800624, rs2070600, and rs184003, with synergistic effects of rs184003 and rs2070600 [87]. The rs184003 SNP was explored in a cohort of patients with idiopathic pulmonary fibrosis, but it was not associated with this phenotype [56]. It is also suggested that rs184003 SNP influences the quantitative insulin sensitivity check index in type 2 diabetic patients [119], and a meta-analysis found an association of rs184003 with increased risk of developing diabetes and diabetic retinopathy in Asian subjects [49].

## 7. Other *AGER* Polymorphisms

### 7.1. rs1051993 NM_001136.4:c.-1435G>T Polymorphism

The G allele frequency in the general population is 93.4% versus 6.6% for allele T. The G allele frequency in the CEU population is 99.5% (0.5% for allele T), and G and T allele frequencies in the EAS population are 91.9% and 8.1%, respectively (1000 Genome data). The potential correlation of rs1051993 SNP with primary open-angle glaucoma related (due to its potential role in oxidative stress), but no significant association has been reported [74].

### 7.2. rs1800684 NM_001136.4:c.6A>T, p.Ala2= polymorphism

The A allele frequency in the general population is 96.4% (3.6% for allele T). The A allele frequency in the CEU population is 84.8% versus 15.2% for allele T. The A and T allele frequencies in the EAS population are 99.2% and 0.8%, respectively (1000 Genome data). The rs1800684 SNP was evaluated in several studies for potential association with increased risk of dementia or Alzheimer's disease, but no significant association was found. No significant association was found with Parkinson disease development in a GWA study. However, an association with increased overall risk of ischemic stroke was reported in women [72, 78, 80, 117, 120, 121].

### 7.3. rs3131300 NM_001136.4:c.52+14T>C Polymorphism

The T allele frequency in the general population is 86.4% (13.6% for allele C). The T allele frequency in the CEU population is 81.8% versus 18.2% for allele C. T and C allele frequencies in the EAS population are 90.6% and 9.4%, respectively (1000 Genome data). No association was found between the rs3131300 SNP and idiopathic pulmonary fibrosis [56], but rs3131300 was in association with diabetic retinopathy in another study [81]. The rs3131300 SNP was associated with neither Alzheimer's disease nor schizophrenia [78, 79].

### 7.4. rs17846804 NM_001136.4:c.83C>T, p.Ala28Val Polymorphism

The C allele frequency in the general population is 99.9% (0.1% for allele T). The C allele frequency in the CEU population is 100%. C and allele frequencies are 99.5% and 0.5%, respectively, in the EAS population (1000 Genome data). Few data on rs17846804 SNP are available, and no association with any pathological risk has been reported to date. Although rs17846804 was found in a cohort of schizophrenic patients, its frequency was the same as in controls [79–81].

### 7.5. rs1035798 NM_001136.4:c.356-57C>T Polymorphism

The C allele frequency in the general population is 85% (15% for allele T). The C allele frequency in the CEU population is 81.3% versus 18.7% for allele T. C and T allele frequencies are 86.1% and 13.9%, respectively, in the EAS population (1000 Genome data). No association was found with idiopathic pulmonary fibrosis [56], but rs1035798 was less present in patients with rheumatoid arthritis than in controls [122]. The rs1035798 SNP was also tested as a risk marker for dementia and Alzheimer's disease, but no significant association was found [78, 120]. The rs1035798 SNP was not associated with schizophrenia [79], but it was associated with increased risk of ischemic stroke in women. The rs1035798 SNP was associated with a mean 24-hour diastolic blood pressure in the GRAPHIC study [123]. It was reported that rs1035798 was associated with small-vessel disease, independently of hypertension, diabetes, and smoking [117]. The rs1035798 SNP was also associated with increased death from cardiovascular events in individuals with common cardiovascular risk factors [26]. An analysis of patients with type 1 diabetes from the Finn Diane cohort reported that rs1035798 was more present in type 1 diabetic patients than in controls [38]. The rs1035798 SNP was tested in a Chinese population for a link between its presence and diabetic retinopathy, but no association was found [124]. In a GWA study in a cohort with high prevalence of diabetes, rs1035798 was, among other SNPs, most associated with AGE levels [36]. The rs1035798 SNP was associated with lower risk of pancreatic cancer in another GWA study [86].

### 7.6. rs17846798 NM_001136.4:c.692-23C>T Polymorphism

The C allele frequency in the general population is 94.6% (5.4% for allele T). The C allele frequency in the CEU population is 99.5% versus 0.5% for allele T. C and T allele frequencies are 96.7% and is 3.3% for allele T (1000 Genome data). In a study about schizophrenia (already reported in the description of rs2071288), rs17846798 was explored in a specific haplotype including rs2071288 and rs184003. Those three SNPs were in perfect linkage disequilibrium. The haplotype was associated with a decrease in esRAGE levels encountered in schizophrenia subjects [79].

### 7.7. rs3134940 NM_001136.4:c.964+208A>G Polymorphism

This SNP is more commonly known as 2184A/G in the literature. The A allele frequency in the general population is 86.7% and is 13.3% for allele G. The A allele frequency in the CEU population is 81.8% and is 18.2% for allele G. The A allele frequency in the EAS population is 90.7% and is 9.3% for allele G (1000 Genome data). The rs3134940 SNP seemed to be in linkage disequilibrium with -429T/C. The rs3134940 SNP is hypothetically located in the regulatory binding site and influences the production of sRAGE [32]. A haplotype analysis including rs1800625 and 2184G had reported that haplotype increases diabetic nephropathy risk in diabetic subjects and was associated with early onset of this phenotype [109]. A study in Ashkenazi or Sephardic Jewish patients with type 1 or type 2 diabetes had shown that rs3134940 was associated with the risk of developing diabetic nephropathy [41]. A study on clear cell renal cancer had shown that rs3134940 was associated with high aggressiveness of the tumor [32]. The rs3134940 SNP was abstracted from a GWA study for pancreatic cancer and was not associated with any risk of pancreatic cancer [86]. The rs3134940 SNP was significantly more prevalent in systemic lupus erythematosus patients and lupus nephritis patients compared with controls. The rs3134940 SNP was also associated with disease severity and response to treatment in lupus nephritis patients [105].

### 7.8. rs2853807 NM_001136.4:c.965-163C>T Polymorphism

The C allele frequency in the general population is 96.3% and is 3.7% for allele T. The C allele frequency in the CEU population is 100%. The C allele frequency in the EAS population is 93% and is 7% for allele T (1000 Genome data). The rs2853807 SNP was explored in a cohort of patients with idiopathic pulmonary fibrosis; it was not associated with this phenotype [56]. Neither was the rs2853807 SNP associated with schizophrenia [79].

### 7.9. rs2071288 NM_001136.4:c.992-6G>A Polymorphism

The G allele frequency in the general population is 94.6% and is 5.4% for allele A. The G allele frequency in the CEU population is 99.5% and is 0.5% for allele A. The G allele frequency in the EAS population is 96.7% and is 3.3% for allele A (1000 Genome data). The rs2071288 SNP located at a splice site in intron 9 was described as being associated with sRAGE and esRAGE levels, probably because of an alternative splicing. This SNP did not show any role in diabetes and cardiovascular risk even when it was involved in variations of sRAGE and esRAGE expressions [125]. In a study about schizophrenia, it was explored in a specific haplotype including rs17846798 and rs184003. Those three SNPs were in perfect linkage disequilibrium. In the haplotype, it was associated with a decrease in esRAGE levels in schizophrenia subjects [79]. In a GWA study, rs2071288 was reported to influence sRAGE levels in Asian type 2 diabetics. sRAGE had shown an important role in diabetic complications such as diabetic kidney disease [20, 48]. Also, in a GWA study on atherosclerosis risk in different communities, rs2071288 was associated with 43% lower sRAGE levels [72]. The rs2071288 SNP was associated with sRAGE levels and diffusing capacity of carbon monoxide in patients with emphysema in COPD. Lower circulating sRAGE levels are associated with emphysema severity [67]. The rs2071288 SNP was explored in a cohort of patients with idiopathic pulmonary fibrosis and was not associated with this phenotype [56].

### 7.10. rs17493811 g.32145399C>G Polymorphism

The C allele frequency in the general population is 98.9% and is 1.1% for allele G. The C allele frequency in the CEU population is 97.5% and is 2.5% for allele G. The C allele frequency in the EAS population is 98.8% and is 1.2% for allele G (1000 Genome data). This SNP is located in the 3′ downstream region of the *AGER* gene. An analysis of patients with type 1 diabetes from the Finn Diane cohort had predicted rs17493811 as increasing the risk of type 1 diabetes [38]. In another study, rs17493811 was not associated with sRAGE levels, which fluctuate and seem to be associated with type 1 diabetes [22].

### 7.11. rs9469089 g.32146657G>C Polymorphism

The G allele frequency in the general population is 86.7% and is 13.3% for allele C. The G allele frequency in the CEU population is 80.8% and is 19.2% for allele C. The G allele frequency in the EAS population is 88.6% and is 11.4% for allele C (1000 Genome data). This SNP is located in the 3′ downstream region of the *AGER* gene. In studies on children with type 1 diabetes, the CC genotype of rs9469089 was linked to increased blood concentrations of sRAGE. It was also described as protective; in fact, rs9469089 was associated with a decreased risk of type 1 diabetes [22, 38].

### 7.12. rs3530981 NM_001136.4:c.143G>A, p.Arg48Gln Polymorphism

Its allele frequency is not available on the 1000 Genome database, perhaps because of its poor frequency. It is a very rare SNP. This SNP has been proposed as a risk predictor only recently [80, 81].

### 7.13. rs35802968 NM_001136.4:c.153G>A p.Trp51Ter Polymorphism

The G allele frequency in the general population is 99.9% and is 0.1% for allele A. The G allele frequency in the CEU population is 100%. The G allele frequency in the EAS population is 100% (1000 Genome data). It is a very rare SNP. The rs35802968 SNP was less studied and did not correlate with any risk of developing any disease [80, 81].

### 7.14. rs35795092 NM_001136.4:c.267C>G, p.Val89= Polymorphism

The C allele frequency in the general population is 93.2% and is 6.8% for allele G. The C allele frequency in the CEU population is 94.9% and is 5.1% for allele G. The C allele frequency in the EAS population is 99.6% and is 0.4% for allele G (1000 Genome data). The rs35795092 SNP was reported in two studies but was never associated with any pathological risk [72, 80].

### 7.15. rs17846806 NM_001136.4:c.341G>A, p.Arg114Gln Polymorphism

Its allele frequency is not available on the 1000 Genome database, perhaps because of its low frequency. The rs17846806 SNP was reported in two studies and was not associated with increased risk of developing diseases [80, 81].

### 7.16. rs2269422 NM_001136.4:c.355+38A>G Polymorphism

The A allele frequency in the general population is 95.8% and is 4.2% for allele G. The A allele frequency in the CEU population is 100%. The A allele frequency in the EAS population is 86.9% and is 13.1% for allele G (1000 Genome data). The rs2269422 SNP was explored in a cohort of patients with idiopathic pulmonary fibrosis but was not associated with this phenotype [56]. The rs2269422 SNP was neither associated with schizophrenia nor with blood pressure nor hypertension [79, 116].

## 8. Linkage Disequilibrium with MHC Gene Variants

As said in the introduction of the *AGER* locus, involved in inflammatory and immune responses is also the locus of major histocompatibility complex (MHC) III. This III region does not have genes involved in antigenic presentation; however, it is between regions I and II including *HLA-A*, *HLA-B* and *HLA-C*, *DPA*, *DPB*, *DQA*, *DQB*, *DRA*, and *DRB1* genes, respectively. All MHC genes possess multiallelic polymorphism, codominance, and haplotype transmission. So, this element could predict that some MHC haplotypes could be in linkage disequilibrium (LD) with some *AGER* gene variants. A few studies have documented this point. A work on rheumatoid arthritis showed that G82S was associated with its disease independently of *HLA DRB11* [127]. In type 1 diabetic patients, LD between *AGER* and *HLA* variants seemed to have a clinical correlation. Notably, the -429C allele was in LD with an ancestral haplotype of HLA and was associated with higher maximal hemoglobin A1C concentration [128]. *AGER* and *DRB1* variants seemed to work in synergy in activation of immune response and in involving diabetes type 1 complications [129]. The S82 *AGER* allele was in LD too with some MHC variants [130]. Finally, a study on Alzheimer's disease also showed that *AGER* and HMC variants were in synergy to be associated in such pathology as it is also the case in chronic fatigue syndrome [131, 132].

## 9. Conclusion

RAGE is a membrane receptor with major roles in many molecular and cellular pathways and disorders. It is crucial for fetal development, especially in the brain and the lungs. RAGE expression fluctuates depending on its environment, and RAGE has pathophysiological implications in various diseases such as neurodegenerative diseases, lung diseases, or cancers. In addition, its circulating isoform sRAGE has been proposed as a biomarker in some diseases. However, the *AGER* gene (encoding RAGE proteins) is a complex gene with many transcripts with complex and incompletely understood regulation. RAGE is a highly polymorphic gene, with around ten polymorphisms being predominantly studied in the available literature. Our review is aimed at providing evidence and perspectives for the implication of RAGE polymorphisms in the onset and severity of diseases. Nevertheless, some studies have contradictory findings and ethnicity may be the key to understanding these apparent contradictions. The true impact of *AGER* polymorphisms remains underinvestigated, and it is crucial to clearly define the origin of the study population. Although RAGE has promising risk prediction and prognostic values in many conditions, further studies are needed to assess the impact of *AGER* polymorphisms in disease pathophysiology.

## Figures and Tables

**Table 1:** Polymorphisms of the *AGER* gene (transcript NM_001136.4) studied in this review and their potential consequences.

rs	Nucleotide change	Amino acid change	Exon/11	1000 genome allele frequency	1000 genome CEU allele frequency	1000 genome EAS allele frequency	Other names	Consequences	Reference
rs2070600	c.244G>A	p.Gly82Ser	3	0.072	0.076	0.219	G82S	Microangiopathy in DT2	[28]
								Diabetic complications	[38]
								Rheumatoid arthritis	[32, 50–52]
								Cancer	[54, 56, 57, 59]
								Lung pathologies such as COPD and ARDS	[69]
								Alzheimer's disease	[75–78]
								Psychoticism factors	[33]

rs1800624	c.-388T>A		Promoter	0.15	0.192	0.137	-374T/A	Reduced risk of heart disease	[29, 89, 93, 97–99]
								Reduced risk of cancer	[29, 50, 53]
								DT1 complications	[38, 126]
								Reduced risk of Crohn's disease	[101, 102]

rs1800625	c.-443T>C		Promoter	0.137	0.182	0.096	-429T/C	Diabetic retinopathy	[32, 108–110]
								Cancer	[32]
								Lupus	[105]
								Protective of cardiovascular risk	[18, 114]

NA	c.-421_-359del		Promoter				63 bp deletion	Reduced survival of heart disease in diabetic nephropathy	[118]

rs184003	c.822+49G>T		Intron 7-8	0.156	0.051	0.146	1704G/T	Coronary artery disease	[115]
								Breast cancer	[87]
								Diabetes	[49]

rs1051993	c.-1435G>T		5′UTR	0.066	0.005	0.081		///	

rs1800684	c.6A>T	p.Ala2=	1	0.036	0.152	0.008		Ischemic stroke in women	[72]

rs3131300	c.52+14T>C		Intron 1-2	0.136	0.182	0.094		Diabetic retinopathy	[81]

rs17846804	c.83C>T	p.Ala28Val	2	0.001	0	0.005		///	

rs3530981	c.143G>A	p.Arg48Gln	2					///	

rs35802968	c.153G>A	p.Trp51Ter	2	0.001	0	0		///	

rs35795092	c.267C>G	p.Val89=	3	0.068	0.051	0.004		///	

rs17846806	c.341G>A	p.Arg114Gln	3					///	

rs2269422	c.355+38A>G		Intron 3-4	0.042	0	0.131		///	

rs1035798	c.356-57C>T		Intron 3-4	0.15	0.187	0.139		Lower risk of rheumatoid arthritis	[122]
								Small-vessel disease	[117]
								DT1	[38]
								Lower risk of pancreatic cancer	[86]

rs17846798	c.692-23C>T		Intron 6-7	0.054	0.005	0.033		///	

rs3134940	c.964+208A>G		Intron 8-9	0.133	0.182	0.093	+2184A/G	Diabetic nephropathy	[41, 109]
								High aggressiveness of tumor in clear cell renal cancer	[32]
								Lupus	[105]

rs2853807	c.965-163C>T		Intron 8-9	0.037	0	0.07	+2245G/A	///	

rs2071288	c.992-6G>A		Intron 9-10	0.054	0.005	0.033		Emphysema in COPD	[67]

rs17493811	g.32177622C>G		3′UTR	0.011	0.025	0.012		DT1	[22, 38]

rs9469089	g.32178880G>A		3′UTR	0.133	0.192	0.114		Decreased risk of DT1	[22, 38]

DT1: type 1 diabetes; DT2: type 2 diabetes; COPD: chronic obstructive pulmonary disease; ARDS: acute respiratory distress syndrome.
